# Crystal structure of 1-[2,4-bis(4-methoxy­phenyl)-3-azabicyclo[3.3.1]nonan-3-yl]ethanone

**DOI:** 10.1107/S1600536814022545

**Published:** 2014-10-24

**Authors:** V. Shreevidhyaa Suressh, S. Sathya, A. Akila, S. Ponnuswamy, G. Usha

**Affiliations:** a Department of Physics, Anna Adarsh College for Women, Chennai-40, Tamilnadu, India; b PG and Research Department of Physics, Queen Mary’s College, Chennai-4, Tamilnadu, India; c PG and Research Department of Chemistry, Government Arts College, Coimbatore-18, Tamilnadu, India

**Keywords:** crystal structure, aza­bicyclo­[3.3.1]nona­ne, cyclo­hexane ring, piperidine ring

## Abstract

In the title compound, C_24_H_29_NO_3_, the aza­bicycle contains two six-membered rings, *viz.* a cyclo­hexane ring and a piperidine ring. The first adopts a chair conformation and the second a half-chair conformation. The dihedral angle between their mean planes is 86.21 (13)°, indicating that they are almost perpendicular to one another. The dihedral angle between the planes of the 4-meth­oxy­phenyl rings is 17.51 (13)°, and they make dihedral angles of 81.9 (3) and 81.3 (3)° with the ethan-1-one group. In the crystal, mol­ecules are linked by C—H⋯π inter­actions forming chains along [10-1].

## Related literature   

For the biological activity of piperidine derivatives, see: Barker *et al.* (2005 ▶); Hardick *et al.* (1996 ▶); Jeyaraman & Avila (1981 ▶); Parthiban, Aridoss *et al.* (2009 ▶); Parthiban, Rathika *et al.* (2010 ▶). For the crystal structures of similar compounds, see: Parthiban *et al.* (2008 ▶); Parthiban, Ramkumar *et al.* (2009 ▶, 2010 ▶).
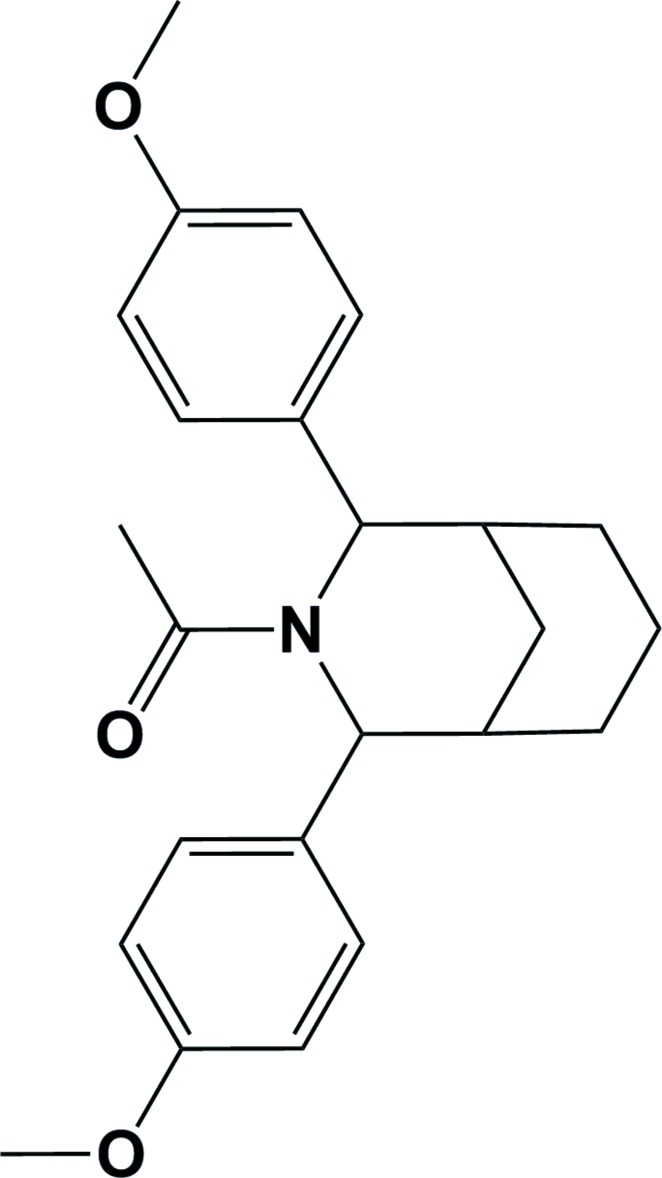


## Experimental   

### Crystal data  

C_24_H_29_NO_3_
*M* *_r_* = 379.48
Monoclinic, 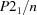

*a* = 7.6309 (13) Å
*b* = 17.102 (3) Å
*c* = 15.395 (2) Å
β = 93.886 (5)°
*V* = 2004.5 (6) Å^3^
*Z* = 4
Mo *K*α radiation
μ = 0.08 mm^−1^
*T* = 293 K
0.35 × 0.30 × 0.25 mm

### Data collection  

Bruker Kappa APEXII CCD diffractometer
Absorption correction: multi-scan (*SADABS*; Bruker, 2008 ▶) *T*_min_ = 0.972, *T*_max_ = 0.980
17249 measured reflections
4597 independent reflections
2524 reflections with *I* > 2σ(*I*)
*R*_int_ = 0.070

### Refinement  

*R*[*F* ^2^ > 2σ(*F* ^2^)] = 0.065
*wR*(*F* ^2^) = 0.267
*S* = 0.86
4597 reflections
253 parameters
H-atom parameters constrained
Δρ_max_ = 0.36 e Å^−3^
Δρ_min_ = −0.31 e Å^−3^

### 

Data collection: *APEX2* (Bruker, 2008 ▶); cell refinement: *SAINT* (Bruker, 2008 ▶); data reduction: *SAINT*; program(s) used to solve structure: *SHELXS97* (Sheldrick, 2008 ▶); program(s) used to refine structure: *SHELXL97* (Sheldrick, 2008 ▶); molecular graphics: *ORTEP-3 for Windows* (Farrugia, 2012 ▶) and *Mercury* (Macrae *et al.*, 2008 ▶); software used to prepare material for publication: *SHELXL97* and *PLATON* (Spek, 2009 ▶).

## Supplementary Material

Crystal structure: contains datablock(s) I, New_Global_Publ_Block. DOI: 10.1107/S1600536814022545/su2798sup1.cif

Structure factors: contains datablock(s) I. DOI: 10.1107/S1600536814022545/su2798Isup2.hkl

Supporting information file. DOI: 10.1107/S1600536814022545/su2798Isup3.cml

**Fig. 1.**
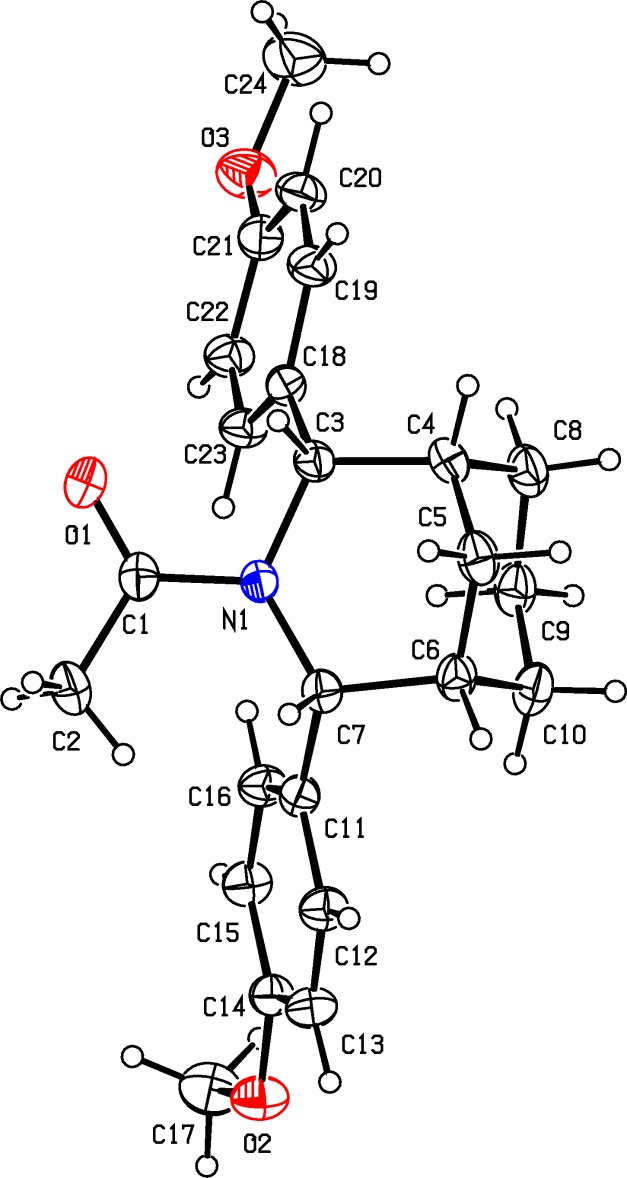
The molecular structure of the title compound, showing the atom labelling. Displacement ellipsoids are drawn at the 30% probability level.

. DOI: 10.1107/S1600536814022545/su2798fig1.tif
The mol­ecular structure of the title compound, showing the atom labelling. Displacement ellipsoids are drawn at the 30% probability level.

**Fig. 2.**
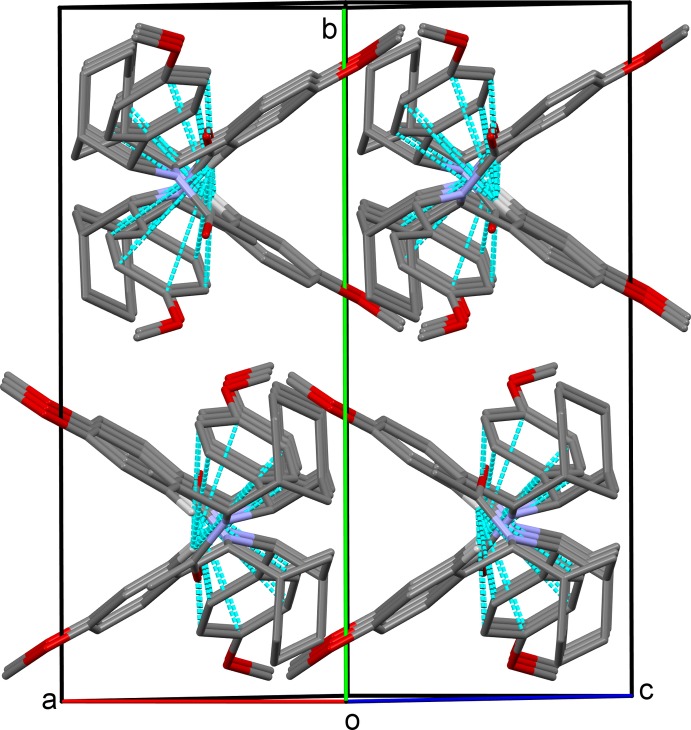
A view of the crystal packing of the title compound, with C—H···π interactions indicted by dashed lines (see Table 1 for details).

. DOI: 10.1107/S1600536814022545/su2798fig2.tif
A view of the crystal packing of the title compound, with C—H⋯π inter­actions indicted by dashed lines (see Table 1 for details).

CCDC reference: 1029084

Additional supporting information:  crystallographic information; 3D view; checkCIF report

## Figures and Tables

**Table 1:** Hydrogen-bond geometry (, ) *Cg* is the centroid of the C18C23 ring.

*D*H*A*	*D*H	H*A*	*D* *A*	*D*H*A*
C12H12*Cg* ^i^	0.93	2.97	3.843(3)	158

Symmetry code: (i) 
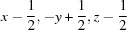
.

## supplementary crystallographic information

### S1. Comment

Azabicyclononanes are present in many alkaloids and they have immense biological activities (Jeyaraman & Avila, 1981; Hardick *et al.*, 1996; Barker *et al.*, 2005). This class of compounds has been observed to exhibit a wide range of biological activities, like antifungal, antibacterial, antimycobacterial, analgesic, antagonistic, anticancer, anti-inflammatory, local anesthetic and hypotensive activity (Parthiban, Aridoss *et al.*, 2009; Parthiban, Rathika *et al.*, 2010).

The molecular structure of the title compound is illustrated in Fig. 1. The cyclohexane ring (C4–C6/C8–C10) adopts a chair conformation [puckering parameters of q2 = 1.3151 (6) Å, T2 = -21.70 (4)°, q3 = 0.0436 (7) Å, QT = 1.3158 (6) Å and φ2 = 88.10 (3)° with the smallest asymmetry parameter D2(C5) = 0.0757 (1)°]. The piperidine ring (C3–C7/N1) adopts a half-chair conformation [puckering parameters of q2 = 0.4230 (5) Å, T2 = 133.78 (5)°, q3 = -0.4053 (5) Å, QT = 0.5858 (5) Å and φ2 = 0.60 (7)° with the smallest asymmetry parameters DS(N1) = 0.0027 (3)° and D2(N1) = 0.2432 (2)°]. Their mean planes are almost normal to one another with a dihedral angle of 86.21 (13)°. The two benzene rings (C11–C16 and C18–C23) are inclined to one another by 17.51 (13)°.

In the crystal, molecules are linked by C—H···π interactions, forming chains along [101] (Table 1 and Fig. 2).

### S2. Experimental

2,4-Bis(4-methoxyphenyl)-3-azabicyclo[3.3.1]nonan-9-one (5 mmol) was dissolved in benzene (80 ml). To this solution, triethylamine (20 mmol) and acetyl chloride (20 mmol) were added and the reaction mixture was stirred using a magnetic stirrer. The precipitated ammonium salt was dried, purified and evaporated. The oily mass was crystallized from petroleum ether (333–353 K).

### S3. Refinement

Crystal data, data collection and structure refinement details are summarized in Table 1. H atoms were positioned geometrically and treated as riding, with C—H = 0.93–0.98 Å and *U*_iso_(H) = 1.5*U*_eq_(C) for methyl H atoms or 1.2*U*_eq_(C) for other H atoms.

### Crystal data

**Table:**

C_24_H_29_NO_3_	*F*(000) = 816
*M**_r_* = 379.48	*D*_x_ = 1.258 Mg m^−^^3^
Monoclinic, *P*2_1_/*n*	Mo *K*α radiation, λ = 0.71073 Å
Hall symbol: -P 2yn	Cell parameters from 4597 reflections
*a* = 7.6309 (13) Å	θ = 1.8–27.5°
*b* = 17.102 (3) Å	µ = 0.08 mm^−^^1^
*c* = 15.395 (2) Å	*T* = 293 K
β = 93.886 (5)°	Block, colourless
*V* = 2004.5 (6) Å^3^	0.35 × 0.30 × 0.25 mm
*Z* = 4	

### Data collection

**Table:**

Bruker Kappa APEXII CCD diffractometer	4597 independent reflections
Radiation source: fine-focus sealed tube	2524 reflections with *I* > 2σ(*I*)
Graphite monochromator	*R*_int_ = 0.070
ω and φ scan	θ_max_ = 27.5°, θ_min_ = 1.8°
Absorption correction: multi-scan (*SADABS*; Bruker, 2008)	*h* = −9→9
*T*_min_ = 0.972, *T*_max_ = 0.980	*k* = −17→22
17249 measured reflections	*l* = −11→19

### Refinement

**Table:**

Refinement on *F*^2^	Primary atom site location: structure-invariant direct methods
Least-squares matrix: full	Secondary atom site location: difference Fourier map
*R*[*F*^2^ > 2σ(*F*^2^)] = 0.065	Hydrogen site location: inferred from neighbouring sites
*wR*(*F*^2^) = 0.267	H-atom parameters constrained
*S* = 0.86	*w* = 1/[σ^2^(*F*_o_^2^) + (0.2*P*)^2^] where *P* = (*F*_o_^2^ + 2*F*_c_^2^)/3
4597 reflections	(Δ/σ)_max_ < 0.001
253 parameters	Δρ_max_ = 0.36 e Å^−^^3^
0 restraints	Δρ_min_ = −0.31 e Å^−^^3^

### Special details

**Table:**

Geometry. All e.s.d.'s (except the e.s.d. in the dihedral angle between two l.s. planes) are estimated using the full covariance matrix. The cell e.s.d.'s are taken into account individually in the estimation of e.s.d.'s in distances, angles and torsion angles; correlations between e.s.d.'s in cell parameters are only used when they are defined by crystal symmetry. An approximate (isotropic) treatment of cell e.s.d.'s is used for estimating e.s.d.'s involving l.s. planes.
Refinement. Refinement of *F*^2^ against ALL reflections. The weighted *R*-factor *wR* and goodness of fit *S* are based on *F*^2^, conventional *R*-factors *R* are based on *F*, with *F* set to zero for negative *F*^2^. The threshold expression of *F*^2^ > σ(*F*^2^) is used only for calculating *R*-factors(gt) *etc*. and is not relevant to the choice of reflections for refinement. *R*-factors based on *F*^2^ are statistically about twice as large as those based on *F*, and *R*- factors based on ALL data will be even larger.

### Fractional atomic coordinates and isotropic or equivalent isotropic displacement parameters (Å^2^)

**Table:**

	*x*	*y*	*z*	*U*_iso_*/*U*_eq_	
C17	0.8638 (4)	0.0399 (2)	−0.3303 (2)	0.0653 (9)	
H17A	0.9081	0.0196	−0.3826	0.098*	
H17B	0.8454	−0.0023	−0.2909	0.098*	
H17C	0.9472	0.0760	−0.3034	0.098*	
O2	0.7019 (3)	0.07911 (13)	−0.35081 (12)	0.0573 (6)	
C1	0.5412 (3)	0.29402 (17)	0.00731 (18)	0.0436 (7)	
C2	0.6109 (4)	0.32952 (18)	−0.0732 (2)	0.0568 (8)	
H2A	0.5557	0.3049	−0.1240	0.085*	
H2B	0.7356	0.3217	−0.0721	0.085*	
H2C	0.5857	0.3845	−0.0748	0.085*	
C3	0.3379 (3)	0.21999 (15)	0.08463 (15)	0.0374 (6)	
H3	0.3145	0.2716	0.1091	0.045*	
C4	0.1578 (3)	0.17762 (16)	0.07593 (18)	0.0433 (7)	
H4	0.0932	0.1932	0.1261	0.052*	
C5	0.0536 (3)	0.20673 (18)	−0.00516 (18)	0.0484 (7)	
H5A	0.0475	0.2634	−0.0046	0.058*	
H5B	−0.0652	0.1863	−0.0071	0.058*	
C6	0.1460 (3)	0.17879 (15)	−0.08472 (18)	0.0427 (7)	
H6	0.0733	0.1948	−0.1366	0.051*	
C7	0.3238 (3)	0.22232 (15)	−0.08655 (16)	0.0367 (6)	
H7	0.2947	0.2745	−0.1095	0.044*	
C8	0.1669 (4)	0.08798 (17)	0.07604 (19)	0.0515 (8)	
H8A	0.2353	0.0706	0.1279	0.062*	
H8B	0.0492	0.0670	0.0781	0.062*	
C9	0.2488 (4)	0.05627 (17)	−0.00362 (18)	0.0504 (8)	
H9A	0.2391	−0.0003	−0.0046	0.061*	
H9B	0.3725	0.0697	−0.0009	0.061*	
C10	0.1580 (4)	0.08982 (16)	−0.0861 (2)	0.0514 (7)	
H10A	0.2217	0.0739	−0.1356	0.062*	
H10B	0.0404	0.0683	−0.0939	0.062*	
C11	0.4351 (3)	0.18444 (14)	−0.15337 (16)	0.0369 (6)	
C12	0.3798 (4)	0.19133 (16)	−0.24134 (17)	0.0445 (7)	
H12	0.2807	0.2210	−0.2573	0.053*	
C13	0.4695 (4)	0.15478 (18)	−0.30543 (17)	0.0485 (7)	
H13	0.4288	0.1591	−0.3636	0.058*	
C14	0.6198 (4)	0.11180 (16)	−0.28286 (17)	0.0418 (6)	
C15	0.6765 (4)	0.10439 (16)	−0.19611 (17)	0.0453 (7)	
H15	0.7769	0.0756	−0.1802	0.054*	
C16	0.5828 (3)	0.14024 (16)	−0.13281 (17)	0.0433 (7)	
H16	0.6212	0.1341	−0.0746	0.052*	
C18	0.4612 (3)	0.17922 (15)	0.15212 (16)	0.0374 (6)	
C19	0.4146 (4)	0.17657 (17)	0.23760 (17)	0.0468 (7)	
H19	0.3166	0.2046	0.2526	0.056*	
C20	0.5081 (4)	0.13399 (18)	0.30143 (17)	0.0490 (7)	
H20	0.4711	0.1326	0.3577	0.059*	
C21	0.6567 (4)	0.09341 (16)	0.28126 (17)	0.0453 (7)	
C22	0.7103 (4)	0.09808 (17)	0.19684 (18)	0.0469 (7)	
H22	0.8128	0.0730	0.1828	0.056*	
C23	0.6124 (3)	0.13963 (16)	0.13384 (17)	0.0428 (7)	
H23	0.6492	0.1410	0.0775	0.051*	
C24	0.6961 (5)	0.0322 (2)	0.4205 (2)	0.0767 (11)	
H24A	0.7826	0.0026	0.4546	0.115*	
H24B	0.5899	0.0022	0.4123	0.115*	
H24C	0.6724	0.0800	0.4502	0.115*	
N1	0.4169 (3)	0.23550 (12)	−0.00014 (12)	0.0360 (5)	
O1	0.5968 (3)	0.31891 (13)	0.07875 (14)	0.0597 (6)	
O3	0.7598 (3)	0.04949 (14)	0.33867 (13)	0.0630 (6)	

### Atomic displacement parameters (Å^2^)

**Table:**

	*U*^11^	*U*^22^	*U*^33^	*U*^12^	*U*^13^	*U*^23^
C17	0.070 (2)	0.064 (2)	0.064 (2)	0.0193 (17)	0.0146 (17)	−0.0021 (16)
O2	0.0631 (13)	0.0652 (14)	0.0442 (11)	0.0102 (11)	0.0079 (10)	−0.0055 (10)
C1	0.0374 (15)	0.0421 (15)	0.0515 (17)	−0.0049 (12)	0.0034 (12)	−0.0017 (13)
C2	0.0556 (18)	0.0518 (18)	0.064 (2)	−0.0179 (14)	0.0117 (15)	0.0049 (14)
C3	0.0374 (14)	0.0358 (14)	0.0397 (14)	−0.0005 (11)	0.0086 (11)	−0.0041 (11)
C4	0.0357 (14)	0.0470 (16)	0.0483 (16)	−0.0040 (12)	0.0115 (12)	0.0034 (12)
C5	0.0313 (14)	0.0492 (17)	0.0648 (19)	0.0001 (12)	0.0032 (13)	0.0075 (14)
C6	0.0338 (14)	0.0456 (16)	0.0477 (15)	−0.0032 (12)	−0.0055 (11)	0.0053 (12)
C7	0.0355 (13)	0.0336 (14)	0.0407 (14)	0.0011 (11)	0.0011 (11)	0.0041 (10)
C8	0.0488 (16)	0.0467 (17)	0.0588 (18)	−0.0121 (13)	0.0016 (14)	0.0132 (14)
C9	0.0506 (17)	0.0371 (16)	0.063 (2)	−0.0080 (13)	−0.0009 (14)	0.0040 (13)
C10	0.0493 (17)	0.0435 (17)	0.0601 (18)	−0.0119 (13)	−0.0054 (14)	−0.0028 (14)
C11	0.0377 (14)	0.0348 (14)	0.0378 (13)	−0.0012 (11)	−0.0009 (11)	0.0025 (11)
C12	0.0440 (15)	0.0485 (17)	0.0401 (14)	0.0068 (13)	−0.0048 (12)	0.0055 (12)
C13	0.0553 (17)	0.0549 (18)	0.0345 (14)	0.0035 (14)	−0.0017 (12)	0.0024 (12)
C14	0.0448 (15)	0.0432 (15)	0.0376 (13)	−0.0006 (12)	0.0041 (11)	−0.0011 (11)
C15	0.0422 (15)	0.0488 (17)	0.0445 (15)	0.0091 (13)	−0.0001 (12)	0.0004 (12)
C16	0.0428 (15)	0.0502 (16)	0.0363 (13)	0.0056 (13)	−0.0023 (11)	0.0026 (12)
C18	0.0382 (14)	0.0371 (14)	0.0374 (13)	−0.0039 (11)	0.0063 (11)	−0.0023 (10)
C19	0.0407 (15)	0.0607 (19)	0.0400 (15)	0.0036 (13)	0.0100 (12)	−0.0045 (13)
C20	0.0486 (16)	0.0635 (19)	0.0361 (14)	0.0015 (14)	0.0112 (12)	0.0015 (13)
C21	0.0458 (15)	0.0460 (16)	0.0437 (15)	−0.0018 (13)	−0.0002 (12)	0.0041 (12)
C22	0.0440 (15)	0.0549 (18)	0.0426 (15)	0.0072 (13)	0.0085 (12)	−0.0051 (13)
C23	0.0441 (15)	0.0490 (16)	0.0359 (13)	0.0041 (13)	0.0080 (11)	−0.0029 (12)
C24	0.084 (2)	0.090 (3)	0.056 (2)	0.001 (2)	−0.0027 (18)	0.0272 (19)
N1	0.0338 (11)	0.0365 (12)	0.0376 (11)	−0.0050 (9)	0.0028 (9)	0.0016 (9)
O1	0.0614 (14)	0.0608 (14)	0.0563 (13)	−0.0233 (11)	0.0008 (10)	−0.0103 (10)
O3	0.0631 (14)	0.0753 (16)	0.0499 (12)	0.0115 (12)	−0.0012 (10)	0.0149 (11)

### Geometric parameters (Å, º)

**Table:**

C17—O2	1.423 (4)	C9—C10	1.518 (4)
C17—H17A	0.9600	C9—H9A	0.9700
C17—H17B	0.9600	C9—H9B	0.9700
C17—H17C	0.9600	C10—H10A	0.9700
O2—C14	1.374 (3)	C10—H10B	0.9700
C1—O1	1.228 (3)	C11—C16	1.376 (4)
C1—N1	1.378 (3)	C11—C12	1.396 (3)
C1—C2	1.509 (4)	C12—C13	1.387 (4)
C2—H2A	0.9600	C12—H12	0.9300
C2—H2B	0.9600	C13—C14	1.387 (4)
C2—H2C	0.9600	C13—H13	0.9300
C3—N1	1.498 (3)	C14—C15	1.381 (4)
C3—C18	1.522 (4)	C15—C16	1.390 (4)
C3—C4	1.552 (3)	C15—H15	0.9300
C3—H3	0.9800	C16—H16	0.9300
C4—C5	1.518 (4)	C18—C23	1.383 (4)
C4—C8	1.535 (4)	C18—C19	1.387 (3)
C4—H4	0.9800	C19—C20	1.382 (4)
C5—C6	1.531 (4)	C19—H19	0.9300
C5—H5A	0.9700	C20—C21	1.383 (4)
C5—H5B	0.9700	C20—H20	0.9300
C6—C10	1.524 (4)	C21—O3	1.367 (3)
C6—C7	1.549 (4)	C21—C22	1.391 (4)
C6—H6	0.9800	C22—C23	1.380 (4)
C7—N1	1.482 (3)	C22—H22	0.9300
C7—C11	1.522 (3)	C23—H23	0.9300
C7—H7	0.9800	C24—O3	1.412 (4)
C8—C9	1.514 (4)	C24—H24A	0.9600
C8—H8A	0.9700	C24—H24B	0.9600
C8—H8B	0.9700	C24—H24C	0.9600
			
O2—C17—H17A	109.5	C8—C9—H9B	109.5
O2—C17—H17B	109.5	C10—C9—H9B	109.5
H17A—C17—H17B	109.5	H9A—C9—H9B	108.1
O2—C17—H17C	109.5	C6—C10—C9	113.0 (2)
H17A—C17—H17C	109.5	C6—C10—H10A	109.0
H17B—C17—H17C	109.5	C9—C10—H10A	109.0
C14—O2—C17	117.2 (2)	C6—C10—H10B	109.0
O1—C1—N1	121.4 (2)	C9—C10—H10B	109.0
O1—C1—C2	118.5 (3)	H10A—C10—H10B	107.8
N1—C1—C2	120.2 (2)	C16—C11—C12	117.3 (2)
C1—C2—H2A	109.5	C16—C11—C7	124.3 (2)
C1—C2—H2B	109.5	C12—C11—C7	118.3 (2)
H2A—C2—H2B	109.5	C13—C12—C11	121.4 (2)
C1—C2—H2C	109.5	C13—C12—H12	119.3
H2A—C2—H2C	109.5	C11—C12—H12	119.3
H2B—C2—H2C	109.5	C12—C13—C14	120.0 (2)
N1—C3—C18	114.16 (19)	C12—C13—H13	120.0
N1—C3—C4	114.5 (2)	C14—C13—H13	120.0
C18—C3—C4	110.3 (2)	O2—C14—C15	124.7 (2)
N1—C3—H3	105.7	O2—C14—C13	115.9 (2)
C18—C3—H3	105.7	C15—C14—C13	119.4 (2)
C4—C3—H3	105.7	C14—C15—C16	119.7 (2)
C5—C4—C8	110.5 (2)	C14—C15—H15	120.2
C5—C4—C3	109.2 (2)	C16—C15—H15	120.2
C8—C4—C3	115.2 (2)	C11—C16—C15	122.2 (2)
C5—C4—H4	107.2	C11—C16—H16	118.9
C8—C4—H4	107.2	C15—C16—H16	118.9
C3—C4—H4	107.2	C23—C18—C19	116.6 (2)
C4—C5—C6	108.2 (2)	C23—C18—C3	124.8 (2)
C4—C5—H5A	110.1	C19—C18—C3	118.5 (2)
C6—C5—H5A	110.1	C20—C19—C18	122.6 (3)
C4—C5—H5B	110.1	C20—C19—H19	118.7
C6—C5—H5B	110.1	C18—C19—H19	118.7
H5A—C5—H5B	108.4	C19—C20—C21	119.8 (2)
C10—C6—C5	110.7 (2)	C19—C20—H20	120.1
C10—C6—C7	115.2 (2)	C21—C20—H20	120.1
C5—C6—C7	108.4 (2)	O3—C21—C22	116.3 (2)
C10—C6—H6	107.4	O3—C21—C20	125.1 (3)
C5—C6—H6	107.4	C22—C21—C20	118.6 (3)
C7—C6—H6	107.4	C23—C22—C21	120.4 (3)
N1—C7—C11	114.6 (2)	C23—C22—H22	119.8
N1—C7—C6	115.1 (2)	C21—C22—H22	119.8
C11—C7—C6	109.7 (2)	C18—C23—C22	121.9 (2)
N1—C7—H7	105.5	C18—C23—H23	119.1
C11—C7—H7	105.5	C22—C23—H23	119.1
C6—C7—H7	105.5	O3—C24—H24A	109.5
C9—C8—C4	112.2 (2)	O3—C24—H24B	109.5
C9—C8—H8A	109.2	H24A—C24—H24B	109.5
C4—C8—H8A	109.2	O3—C24—H24C	109.5
C9—C8—H8B	109.2	H24A—C24—H24C	109.5
C4—C8—H8B	109.2	H24B—C24—H24C	109.5
H8A—C8—H8B	107.9	C1—N1—C7	118.1 (2)
C8—C9—C10	110.6 (3)	C1—N1—C3	111.8 (2)
C8—C9—H9A	109.5	C7—N1—C3	123.93 (19)
C10—C9—H9A	109.5	C21—O3—C24	118.3 (2)
			
N1—C3—C4—C5	−36.4 (3)	C12—C11—C16—C15	0.9 (4)
C18—C3—C4—C5	−166.8 (2)	C7—C11—C16—C15	177.6 (2)
N1—C3—C4—C8	88.6 (3)	C14—C15—C16—C11	−1.0 (4)
C18—C3—C4—C8	−41.8 (3)	N1—C3—C18—C23	−17.5 (4)
C8—C4—C5—C6	−60.1 (3)	C4—C3—C18—C23	113.1 (3)
C3—C4—C5—C6	67.6 (3)	N1—C3—C18—C19	167.0 (2)
C4—C5—C6—C10	59.2 (3)	C4—C3—C18—C19	−62.4 (3)
C4—C5—C6—C7	−68.1 (3)	C23—C18—C19—C20	−2.9 (4)
C10—C6—C7—N1	−87.1 (3)	C3—C18—C19—C20	173.0 (3)
C5—C6—C7—N1	37.6 (3)	C18—C19—C20—C21	1.6 (5)
C10—C6—C7—C11	43.8 (3)	C19—C20—C21—O3	179.9 (3)
C5—C6—C7—C11	168.5 (2)	C19—C20—C21—C22	1.3 (4)
C5—C4—C8—C9	58.0 (3)	O3—C21—C22—C23	178.5 (2)
C3—C4—C8—C9	−66.3 (3)	C20—C21—C22—C23	−2.8 (4)
C4—C8—C9—C10	−52.3 (3)	C19—C18—C23—C22	1.4 (4)
C5—C6—C10—C9	−56.1 (3)	C3—C18—C23—C22	−174.2 (2)
C7—C6—C10—C9	67.4 (3)	C21—C22—C23—C18	1.4 (4)
C8—C9—C10—C6	51.7 (3)	O1—C1—N1—C7	164.8 (2)
N1—C7—C11—C16	22.7 (3)	C2—C1—N1—C7	−14.6 (4)
C6—C7—C11—C16	−108.5 (3)	O1—C1—N1—C3	11.4 (4)
N1—C7—C11—C12	−160.7 (2)	C2—C1—N1—C3	−168.1 (2)
C6—C7—C11—C12	68.1 (3)	C11—C7—N1—C1	72.4 (3)
C16—C11—C12—C13	0.3 (4)	C6—C7—N1—C1	−159.1 (2)
C7—C11—C12—C13	−176.5 (2)	C11—C7—N1—C3	−137.6 (2)
C11—C12—C13—C14	−1.5 (4)	C6—C7—N1—C3	−9.1 (3)
C17—O2—C14—C15	−5.0 (4)	C18—C3—N1—C1	−71.7 (3)
C17—O2—C14—C13	175.2 (3)	C4—C3—N1—C1	159.8 (2)
C12—C13—C14—O2	−178.7 (2)	C18—C3—N1—C7	136.7 (2)
C12—C13—C14—C15	1.5 (4)	C4—C3—N1—C7	8.2 (3)
O2—C14—C15—C16	179.9 (3)	C22—C21—O3—C24	−169.6 (3)
C13—C14—C15—C16	−0.2 (4)	C20—C21—O3—C24	11.8 (4)

### Hydrogen-bond geometry (Å, º)

Cg is the centroid of the C18–C23 ring.

**Table:**

*D*—H···*A*	*D*—H	H···*A*	*D*···*A*	*D*—H···*A*
C12—H12···*Cg*^i^	0.93	2.97	3.843 (3)	158

Symmetry code: (i) *x*−1/2, −*y*+1/2, *z*−1/2.
