# Supplementary figures and images for: Crystal structure of 1-[2,4-bis(4-methoxy­phenyl)-3-azabicyclo[3.3.1]nonan-3-yl]ethanone
Source: Acta Crystallogr Sect E Struct Rep Online. 2014 Oct 24;70(Pt 11):o1171–2. doi: 10.1107/S1600536814022545 (PMC4257259; doi:10.1107/S1600536814022545)

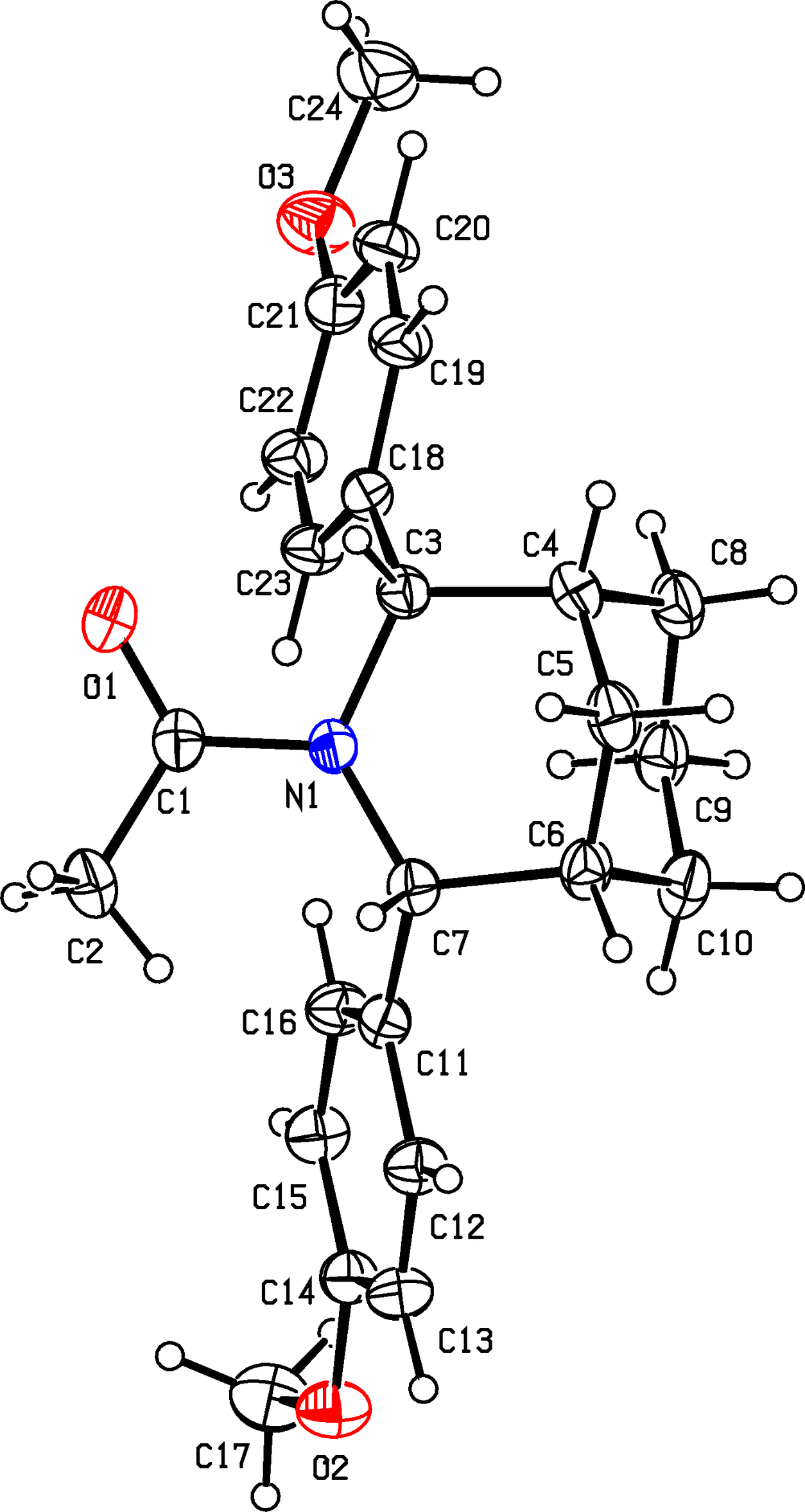

Supplement: Supplementary file 4 [file e-70-o1171-fig1.tif]

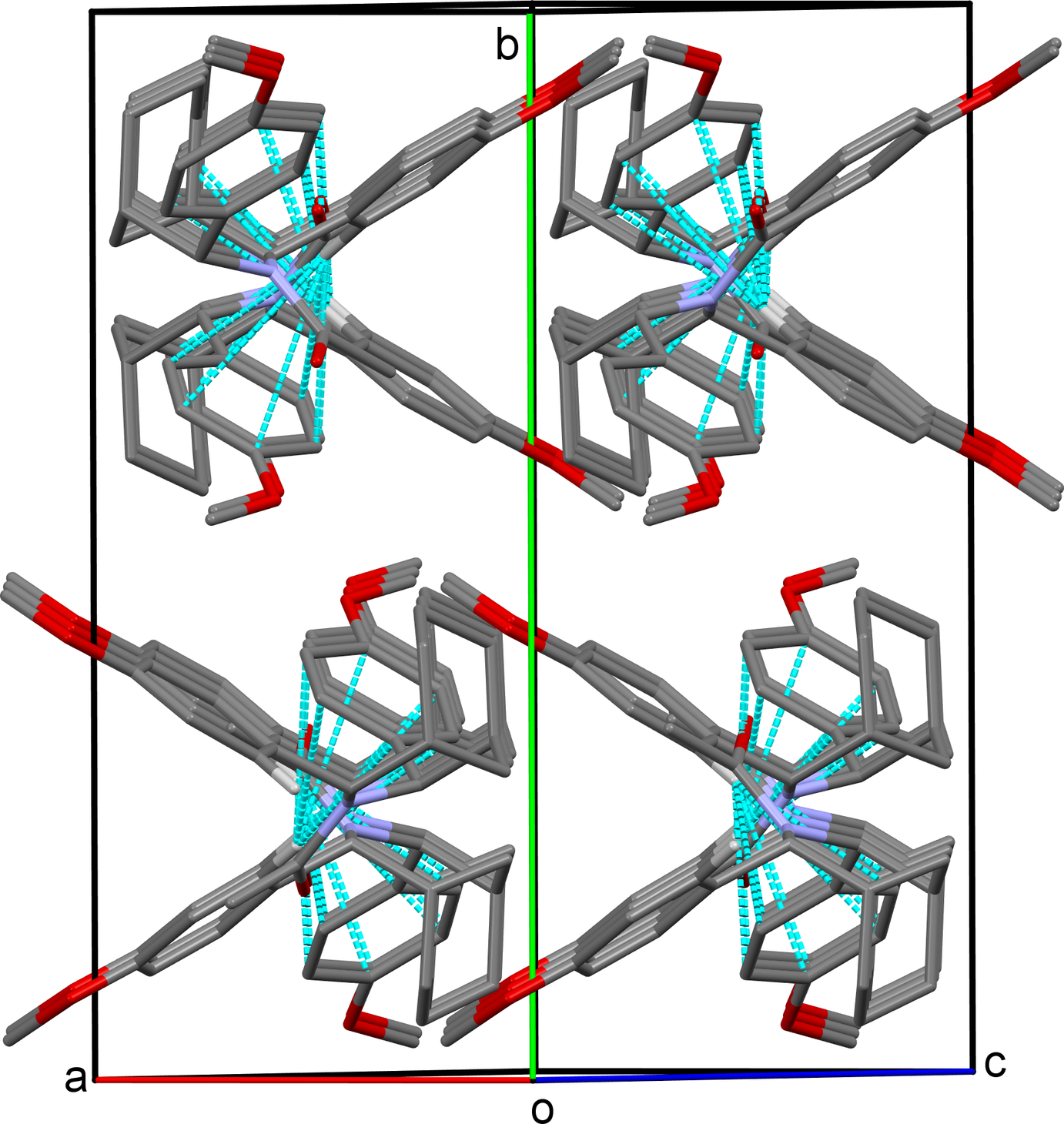

Supplement: Supplementary file 5 [file e-70-o1171-fig2.tif]
